# China and monkeypox: Correspondence

**DOI:** 10.1002/ame2.12279

**Published:** 2022-10-08

**Authors:** Amnuay Keebayoon, Rujittika Mungmunpuntipantip, Viroj Wiwanitkit

**Affiliations:** ^1^ Private Academic Consultant Samraong Cambodia; ^2^ Private Academic Consultant Bangkok Thailand; ^3^ Department of Biological Science Joseph Ayobbabalola University Ikeji‐Arakeji Nigeria

To the editor,

We would like to share our feedback on the publication “Is China ready for monkeypox?”.^1^ Wei documented the wide range of clinical problems associated with monkeypox. Wei mentioned that it is necessary to strengthen the prevention method against monkeypox. Usually, in addition to a fever, the patient with monkeypox has a skin problem, usually vesicula lesions. However, it is possible that neither a fever nor a skin lesion is present in a patient with monkeypox.^2^ According to a recent report by Benites‐Zapata et al., the meta‐analysis results showed that rash occurs in 93% and fever in 72% of patients with monkeypox.^3^ Without the atypical appearance of lesions, the doctor can fail to identify the issue and incorrectly diagnose the disease.^4^ In addition, the general laboratory inquiry is commonly based on the evaluation of crust or vesicle clinical samples. Therefore, it is clear that skin lesion is considered to be a typical clinical problem in almost all published reports. It is important to recognize that some patients display only odd symptoms, such as gastrointestinal and neurological conditions.^5^, ^6^, ^7^

Skin lesions can occasionally present as atypical lesions in certain situations and can be a difficult‐to‐diagnose case. In addition, hemorrhagic vesicles can form and become a hard‐to‐diagnose clinical issue.^2^ Due to the possibility that the patient will not be able to be diagnosed in time, medical management raises the probability that the disease will spread. Atypical sickness signs need to be considered in the current state of disease spread. One of the main obstacles to prevent this new public health problem is negligence toward universal prevention of the disease. It is critical to understand that certain individuals, such as those with neurological and gastrointestinal problems, exhibit only unusual symptoms that might be easily missed.^3^, ^4^, ^5^, ^6^, ^7^ This condition is recognized to have an impact on the neurological, pulmonary, and gastrointestinal systems, as mentioned by Cheema et al.^8^ Although this viral illness is considered to be self‐limiting, its consequences and pandemic potential pose severe public health issues.^8^ The animal model study also confirms the pathological problem in both neurological and gastrointestinal systems in monkeypox.^9^

Atypical skin lesions can occasionally appear in specific clinical circumstances, such as in hidden areas of the body. Wei pointed out that clinical management of biosafety for the monkeypox virus needs to be improved.^1^ As of August 22, 2022, 96 nations, territories, and areas in all 6 World Health Organization (WHO) regions had reported 41 664 laboratory‐confirmed cases of monkeypox and 12 fatalities to the WHO. The reported cases of monkeypox are currently increasing. The disease now spreads from the confined epidemiology pattern in men who have sexual contact with men to the normal heterosexual person of both sexes.^10^, ^11^ The new data need to be updated to successfully control the disease. Contact with an infected person or animal and contact with a contaminated object are some ways the disease can be spread.^10^, ^11^ Lymphadenopathy and a prodromal flu‐like illness are the disease symptoms.^10^, ^11^ It is rare to experience complications such as encephalitis, pneumonitis, eye diseases, or bacterial skin infections.^10^, ^11^ In the most severe case, especially for the case with underlying immunodeficiency problem, the patient might have multiple organ dysfunction and die. It might be difficult to diagnose and classify cases as confirmed, probable, or suspected based on clinical symptoms and laboratory results (Table 1). Polymerase chain reaction is frequently used to confirm the disease.^2^, ^10^, ^11^ At present, some drugs are available to treat orthopoxvirus, which includes monkeypox, but the majority of patients get better with supportive therapy.^10^, ^11^ However, antiviral treatment should be considered for a number of patient populations who are at risk for serious outcomes. It is important to acknowledge the clinical practice of infection control. It is necessary to follow the disease control strategies that are employed in the current COVID‐19 outbreak. The universal prevention of the disease is one of the major strategies to overcome this new public health issue.

**TABLE 1:** Classifying monkeypox cases as confirmed, probable, or suspected based on clinical symptoms and laboratory results

Categories	Details
Suspected	A person who was in contact with a probable or proven monkeypox case in the 21 days preceding the beginning of signs or symptoms and presents with any of the following symptoms: sudden onset of fever (>38.5°C), headache, myalgia (muscle pain/body aches), back pain, extreme weakness, or exhaustion OR an individual who has presented with an unexplained acute skin rash, mucosal lesions, or lymphadenopathy since January 1, 2022 (swollen lymph nodes). Single or several lesions in the anogenital region or elsewhere on the body may be present in the skin rash. Single or many oral, conjunctival, urethral, penile, vaginal, or anorectal lesions are examples of mucosal lesions. Anorectal lesions can also cause inflammation (proctitis), discomfort, and/or bleeding AND in which the following common causes of acute rash or skin lesions do not completely explain the clinical picture: varicella zoster, herpes zoster, measles, herpes simplex, bacterial skin infections, disseminated gonococcus infection, primary or secondary syphilis, chancroid, lymphogranuloma venereum, granuloma inguinale, molluscum contagiosum, allergic reaction
Probable	A patient who has an acute skin rash, mucosal lesions, or lymphadenopathy without reason (swollen lymph nodes). The anogenital region or other parts of the body may develop one or more lesions as part of the skin rash. Single or many oral, conjunctival, urethral, penile, vaginal, or anorectal lesions are examples of mucosal lesions. Anorectal inflammation (proctitis), discomfort, and/or bleeding are additional symptoms of anorectal lesions One or more of the following may be present in the patient: • has an epidemiological link to a probable or confirmed case of monkeypox in the 21 days preceding symptom onset • has had multiple and/or casual sexual partners in the 21 days preceding symptom onset • has detectable levels of anti‐orthopoxvirus (OPXV) IgM antibody2 (during the period of 4–56 days after rash onset); or a fourfold increase in IgG antibody titer based on acute (up to days 5–7) and convalescent (day 21 onward) samples; no recent smallpox/monkeypox vaccination or other confirmed OPXV exposure • has a positive orthopoxviral infection test result (e.g., OPXV‐specific PCR without MPXV‐specific PCR or sequencing)
Confirmed	A confirmed case is a person with a monkeypox infection that has been verified in a laboratory (monkeypox PCR positive)

*Note*: Classification based on September 2022 updated WHO reference (https://www.who.int/emergencies/outbreak‐toolkit/disease‐outbreak‐toolboxes/monkeypox‐outbreak‐toolbox).

According to the current study by Wei, China appears to be well prepared to prevent or control the disease.^1^ Monkeypox has already arrived in our locations in Southeast Asia (such as Singapore and Thailand), near China, and is causing a concern. The majority of new cases are reported in tourists from both African and non‐African nations. Those cases, typically men, easily pass the disease screening at international airports. However, there are also numerous cases of native Chinese people returning from abroad. Those individuals are mainly women working in the services sector abroad. These experiences may provide some guidance for the current situation in China. A simple illness check or simple fever screening and history taking at immigration points may not be sufficient to diagnose the disease. A strict uniform screening method should be implemented to screen everyone (both genders and both foreigners and local Chinese returning from abroad) entering the country. If the number of cases increases rapidly globally, a quarantine for all visitors for a specific period, as employed in COVID‐19 disease control, may be considered in the future.

## AUTHOR CONTRIBUTIONS

AK equal contribution, ideas, writing analyzing, revising, approval for final submissionRM equal contribution, ideas, writing analyzing, revising, approval for final submissionVW equal contribution, ideas, supervising, approval for final submission.

## CONFLICT OF INTEREST

None.
